# Shared feelings? Affective states and transfer in bumblebees

**DOI:** 10.3758/s13420-026-00710-w

**Published:** 2026-02-27

**Authors:** Michael Mendl

**Affiliations:** Animal Welfare and Behaviour Group, Bristol Veterinary School, University of Bristol, Langford House, Langford, BS40 5DU UK

**Keywords:** Emotion, Affective state, Emotional contagion, Bumble bee, Judgement bias

## Abstract

Could bumblebees have emotional states that can be transferred between individuals? A recent study provides positive evidence and illustrates the logic and methods employed to investigate these challenging questions.

The study of animal emotion (affect) is experiencing an explosion of interest (e.g., Adolphs & Anderson, 2018). We have moved from an era in which many ethologists and comparative psychologists were sceptical that emotional states in non-humans (hereafter, animals) could be studied scientifically, or existed at all, to a place where researchers use emotional and affective terms when referring to a range of vertebrate species and, increasingly, to some invertebrates. The notion that such states can be transferred between individuals (‘emotional contagion’), seen as a crucial building block of empathy, is gaining empirical support. A recent paper extends this possibility to an invertebrate, the bumblebee (Romero-Gonzalez et al., 2025), and provides an example of some approaches to address questions that the interested, but perhaps still sceptical, observer may have. How *can* we study emotional states in animals? What do we mean when we say that an animal is in a particular emotional/affective state? How can we infer the transfer of these states between individuals?

Romero-Gonzalez et al. (2025) eschew the use of the word ‘*emotion*’ as being ‘mammal-specific’ and instead opt to refer to ‘*affect*’, a term that is used in the psychological literature to denote internal states that have the property of ‘*valence*’; they are positive or negative, pleasant or unpleasant, rewarding or punishing. These include short-term responses to affectively significant events, technically referred to as ‘*emotions*’ in affective science, and longer-lasting ‘*moods*’ that are not usually attached to any single event.

Because the use of clear terminology matters in an area ripe for misunderstanding, it is relevant to note that ‘mammal-specificity’ is just one reason for being careful when using the term ‘emotion’. Perhaps a more important one is that, due to its colloquial usage, the word is often taken to imply that inferred emotional states are consciously experienced. Whilst there is growing evidence that many vertebrates have conscious experiences, there is less certainty about invertebrates, and there is even evidence for *unconscious* emotion in humans. Therefore, animal emotion researchers should be clear about what they mean by ‘emotion’. Clarity is also needed with respect to how affective constructs can be assessed in animals. To this end, the *componential view* which conceptualises emotional or affective states as comprising behavioural, neurophysiological, cognitive, and conscious ‘components’ is helpful. The first three can be measured objectively in animals, but the conscious component needs to be inferred.

Against this conceptual background, Romero-Gonzalez et al. (2025) chose to use a highly translatable cognitive marker, ‘*judgement bias’*, as their indicator of animal affect. The judgement bias task (JBT) was originally developed for studies of rats (Harding et al., 2004) but has since been used in more than 200 studies of mammals, birds, fish, cephalopods, and insects. It is based on findings from human psychology that people in more negative affective states tend to make more negative (‘pessimistic’) judgements about the likely outcomes of ambiguous stimuli. In evolutionary terms, it also makes sense that individuals in negative states resulting from recent negative experiences could use these states heuristically as Bayesian priors when making decisions about ambiguity, in this case being ‘pessimistically’ cautious in an environment where negative outcomes are likely. ‘Optimistic’ and ‘pessimistic’ decisions under ambiguity should thus reflect relatively positively and negatively valenced affective states respectively, and this is supported by recent meta-analyses (e.g., Lagisz et al., 2020) suggesting that the JBT is a promising indicator of affective valence.

To implement the JBT, Romero-Gonzalez et al. (2025) trained individual bumblebees to forage in an arena containing an array of eight artificial flowers. For each bee, all the flowers were blue during half of the foraging sessions and green during the other half. For 50% of the bees, blue flowers provided a rewarding sucrose solution and green flowers provided unrewarding distilled water; the opposite was true for the other 50%. Bees learned the colour–reward association within eight trials, showing shorter latencies between arena entry and first visit to a flower when flowers were of the rewarded (positive (P)) colour compared with when they were the non-rewarded (negative (N)) colour. Bees were then exposed to five test trials in which all eight flowers were either P, N, or one of three intermediate ambiguous colours (near positive (NP), middle (M), near negative (NN)), based on bee perceptual colour space. Ambiguous colours were not rewarded, and order of testing was carefully balanced across bees.

Prior to a test, manipulations designed to induce affective states were carried out whilst bees were in a tunnel linking the nestbox to the arena. To test the hypothesis that individual experience of reward generates a positive state, bees (*N* = 24 per treatment) were allocated to two groups: *individual reward* (unexpected presentation of a concentrated drop of sucrose solution); *individual control* (no sucrose). Rewarded bees made quicker first visits to ambiguously coloured M flowers than control bees, an ‘optimistic’ bias indicating positive affect, and supporting the hypothesis.

A potential criticism is that quicker visits may have resulted from enhanced arousal and activity following sucrose consumption, rather than positive affect per se. However, this finding was replicated using an active choice JBT variant that didn’t rely on measuring decision latencies. Bees (*N* = 48 per group) were trained in a T-maze in which, for example, a blue-coloured maze was associated with a high-value (50%) sucrose reward in the right arm and unrewarding water in the left arm, and a green maze indicated a low-value (30%) sucrose reward in the left arm and water in the right arm. In this example, positive affect would be indicated by an ‘optimistic’ choice for the right arm when faced with an ambiguous maze colour. This is what was found for rewarded bees compared with controls, ruling out a non-specific treatment effect on activity levels since activity *per se* did not influence readouts in this task.

Romero-Gonzalez et al. (2025) then investigated affective contagion using five further manipulations (*N* = 24 per treatment) in which, prior to testing, the subject bee met another bee whose affective state had been manipulated: *social reward* (encounter with a rewarded bee who had just consumed sucrose); *social control* (encounter with a non-rewarded bee); *short-partition visual reward* (non-contact interaction with a rewarded bee through a short transparent partition); *long-partition visual reward* (as for *short-partition* but using a longer partition forcing bees close to the partition); *non-visual reward* (as for *social reward* but in the dark). *Social reward* and *long-partition visual reward* bees were as quick to visit ambiguous M flowers as *individual reward* bees, and quicker (more ‘optimistic’) than those in the other treatment groups, including *individual* and *social controls*, consistent with the possibility that, under certain conditions, positive affect in one bee can influence that of another.

These findings provide the first evidence of affective contagion in an invertebrate. Specifically, they show that a ‘demonstrator’ bee in a sucrose-rewarded state, known to generate an ‘optimistic’ judgement bias indicative of positive affect can, following a 30-s interaction, induce the same bias in an ‘observer’ bee. The well-designed internal logic of the study directs us to the conclusion that the observer bee is now also in a positive affective state, paralleling similar findings of affective contagion in studies of ravens and rats using the JBT. The study also adds valuable new information by focusing on positive state contagion which has been less studied than that of negative states.

The mechanisms underlying these effects remain to be discovered. Lack of contagion in the *non-visual reward* group indicates that direct transmission of chemosensory information is not sufficient and, together with observed contagion in the *long-partition visual reward* group, suggests that visual information may play an important role. However, absence of contagion in the *short-partition visual reward* group is difficult to reconcile with this explanation unless, as the authors contend, bees need to closely view each other and the long-partition design does indeed force them to spend more time in close proximity. The authors’ suggestion could be tested by comparing the time that bees spent close together in the short- and long-partition groups.

Although more work is needed to elucidate underlying mechanisms, this new study provides further evidence of the affective capabilities of bumblebees. The question of whether bees and other invertebrates consciously experience inferred affective states, and hence are sentient, remains the subject of debate. The outcome will have important implications since it is widely recognised that the capacity for sentience underpins our moral obligation to protect and improve animal welfare (e.g., Birch, 2024).

## References

[CR1] Adolphs, R., & Anderson, D. J. (2018). *The neuroscience of emotion: A new synthesis*. Princeton University Press.

[CR2] Birch, J. (2024). *The edge of sentience*. Oxford University Press.

[CR3] Harding, E. J., Paul, E. S., & Mendl, M. (2004). Cognitive bias and affective state. *Nature,**427*, 312. 14737158 10.1038/427312a

[CR4] Lagisz, M., Zidar, J., Nakagawa, S., Neville, V., Sorato, E., Paul, E. S., ..., & Lovlie, H. (2020). Optimism, pessimism and judgement bias in animals: A systematic review and meta-analysis. *Neuroscience and Biobehavioral Reviews*, *118*, 3–17. 10.1016/j.neubiorev.2020.07.012 32682742

[CR5] Romero-Gonzalez, J., Zho, Z., Chen, L., Peng, C., Solvi, C., & Peng, F. (2025). Positive affective contagion in bumble bees. *Science,**390*, 377–380. 41129631 10.1126/science.adr0216

